# Zero-Inflated Time Series Modelling of COVID-19 Deaths in Ghana

**DOI:** 10.1155/2021/5543977

**Published:** 2021-04-30

**Authors:** Kassim Tawiah, Wahab Abdul Iddrisu, Killian Asampana Asosega

**Affiliations:** Department of Mathematics and Statistics, University of Energy and Natural Resources, Sunyani, Ghana

## Abstract

Discrete count time series data with an excessive number of zeros have warranted the development of zero-inflated time series models to incorporate the inflation of zeros and the overdispersion that comes with it. In this paper, we investigated the characteristics of the trend of daily count of COVID-19 deaths in Ghana using zero-inflated models. We envisaged that the trend of COVID-19 deaths per day in Ghana portrays a general increase from the onset of the pandemic in the country to about day 160 after which there is a general decrease onward. We fitted a zero-inflated Poisson autoregressive model and zero-inflated negative binomial autoregressive model to the data in the partial-likelihood framework. The zero-inflated negative binomial autoregressive model outperformed the zero-inflated Poisson autoregressive model. On the other hand, the dynamic zero-inflated Poisson autoregressive model performed better than the dynamic negative binomial autoregressive model. The predicted new death based on the zero-inflated negative binomial autoregressive model indicated that Ghana's COVID-19 death per day will rise sharply few days after 30^th^ November 2020 and drastically fall just as in the observed data.

## 1. Introduction

Ghana confirmed its first two cases of the novel coronavirus disease on 12^th^ March 2020 at the Noguchi Memorial Institute for Medical Research (NMIMR) [1]. The two cases were all imported. Since then, the government through the Ministry of Health (MoH), the Ghana Health Service (GHS), and other stakeholders introduced prudent measures to help curb the spread of the virus [2]. Key among them was the introduction of the mandatory quarantine of all travellers arriving at the Kotoka International Airport for testing. There was the implementation of social distancing protocols and the compulsory wearing of face/nose masks. Enhanced contact tracing of infected persons and routine surveillance was also instituted. All boarders were subsequently closed to travellers. A partial lockdown of Greater Accra and Greater Kumasi was instituted since they were the hot spots. There was ban on all social gatherings. This led to the closure of all public and private schools, night clubs, and churches. Funerals, weddings, and festivals followed suit. However, private funerals and weddings with a maximum of 25 people with strict adherence to social distancing protocols were permitted. All public transport operators were mandated to reduce their passenger intake in line with the social distancing protocols. The President of Ghana signed an Executive Instrument (EI) to back the ban on social gatherings after Parliament of Ghana passed the Imposition of Restrictions Bill on 21^st^ March 2020. There was also the compulsory washing of hands with soap under running water and use of hand sanitizers. Soap and tissue were placed beside Veronica buckets containing water at vantage points throughout the country to enable people wash their hands frequently with soap under running water after every transaction and engagement. Hugging and handshaking were discouraged. The Government introduced stimulus packages for all frontline health workers to boost their efforts in the fight against the pandemic. The Government also cushioned the entire population with free water since water is key to the fight against the spread of SARS-CoV-2. There was fifty percent electricity subsidy for all consumers. Lifeline consumers of electricity were given hundred percent subsidies. These freebies were initially for a period of 1^st^ April 2020 to 30^th^ June 2020 but were extended for another 3 months [3, 4].

The partial lockdown of Greater Accra and Greater Kumasi was lifted after almost a month or so in operation. Subsequently, social gathering protocols were relaxed to hundred individuals. All public and private schools were reopened to final years to enable them write their final-year examinations. Restrictions on public transport were also lifted. Restrictions on internal public and private transports on land, sea, and air were lifted. Ghana subsequently opened its international airport to foreign travels from 1^st^ September 2020, with strict testing and quarantine rules. Testing results for SARS-CoV-2, the virus that causes COVID-19, 3 weeks prior to arrival at the airport and subsequent testing on arrival were put in place.

Even though the government is making frantic efforts to curb the spread of the coronavirus among the population, the infection figures continue to soar. This calls for an investigation into existing measures and protocols so as to assess their true impact on curtailing the spread of the SARS-CoV-2, the virus that causes COVID-19. Inasmuch as the infection figures are rising, recovery from COVID-19 in Ghana is very impressive.

Maleki et al. [5] purported that for COVID-19 data set, error distribution can cogitate about a two-piece scale mixture of normal (TP-SMN) and designed time series models that work better than ordinary Gaussian and symmetry models. Three regression models (i.e., linear, logarithmic, and quadratic) were proposed for COVID-19 deaths in Pakistan. Influenced by the phase reached by COVID-19 deaths and criteria for assessing goodness of fit, the quadratic model was selected as the best for modelling and predicting death cases in Pakistan [6]. Sperrin and McMillan [4] developed the QCOVID model to predict the risk of COVID-19-related mortality, while the Institute for Health Metrics Evaluation (IHME) proposed and applied deterministic susceptible, exposed, infectious, and recovered (SEIR) compartment frame work model for cases in the United States of America [7].

Dwomoh et al. [8] used mathematical models to investigate COVID-19 infection dynamics in Ghana and delivered a brief forecast of the pandemic trajectory in the country using generalized growth models. They investigated the effective basic reproduction number of the virus in real time applying different techniques of estimation, thereby predicting worse case scenarios amidst integrated individual and Government interventions by the use of compartmental models. Their result indicated that improved individual-level intervention and intensified media coverage can substantially suppress COVID-19 transmission in Ghana and as a result reduce the COVID-19 death rates in the country. However, there seem to be a rise in the daily infection amidst increased Government and media coverage with reduced individual-level intervention. This rise in infections has increased the daily COVID-19-related deaths.

With COVID-19 data from Ghana and Egypt, Asamoah et al. [9] applied sensitivity analysis to suggest that increased diagnoses, enhanced contact tracing, and stringent safety protocols in hospitals or isolation centers with constant supply of PPEs will help reduce (or possibly stop) the spread of the virus in the two countries.

Bonful et al. [3] audited forty-five public transport stations in the Greater Accra region of Ghana to assess the compliance with the World Health Organization (WHO) safety protocols on the prevention of the spread of COVID-19. These included hand hygiene assessment scale, the availability and use of hand washing facilities, social distancing, and on-going public education on COVID-19 prevention measures. Their findings revealed inadequate washing places, lack of public education on practicing personal hygiene, inadequate alcohol-based sanitizers, and improper face-mask wearing (or no face-mask wearing). They concluded that there is a challenge with COVID-19 prevention compliance.

In this paper, we investigated the current trend of COVID-19 mortalities and use it to predict possible future COVID-19 death trajectory so as to help policy makers to readjust their interventions and strategies. The findings will also help individuals improve their quest to fight the spread of the virus to reduce the number of deaths related to the pandemic. It will also push the media to intensity their coverage to create awareness on what the future holds in relation to possible human life's likely to be lost to COVID-19.

We explored zero-inflated time series models [10–15] to Ghana's COVID-19 death counts per update. The foundation for modelling count data with repeated zeros and overdispersion was provided by Lambert [16], Lee et al. [17], Laird and Ware [18], Min and Agresti [19], Ridout et al. [20], and Yau et al. [21]. Zhu [22] proposed zero-inflated Poisson and negative binomial integer-valued generalized autoregressive conditional heteroscedastic (INGARCH) models, while Yang [14] and Yang et al. [15] proposed a zero-inflated Poisson and negative binomial autoregressive models for zero-inflated and overdispersed discrete count time series data.

We employed zero-inflated time series models as proposed by Yang [14] and Yang et al. [15] as it was best suited for our data. The befitting model obtained was used to predict COVID-19 deaths in Ghana in order to assist the Government and public health experts who are managing the pandemic to know what to expect in terms of deaths before they occur so as to plan ahead.

## 2. Materials and Methods

### 2.1. Materials

**Figure 1:**
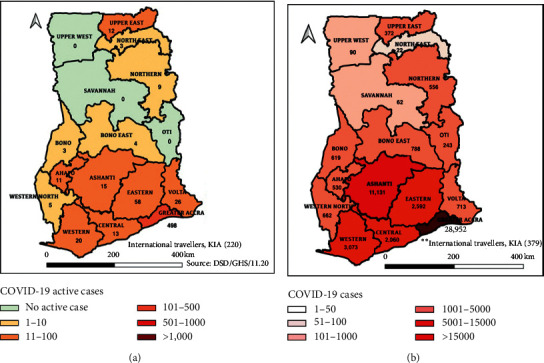
Map of study setting (source: https://www.ghanahealthservice.org/covid19/archive.php#).

We used the entire Ghana as the study setting.  Figure 1 presents the map of the study setting showing the number of active and cumulative confirmed COVID-19 cases as at 22^nd^ November 2020 in each of the 16 administrative regions of Ghana.

The data for this study consist of confirmed COVID-19 deaths per day from 13^th^ March 2020 to 22^nd^ November 2020. The daily COVID-19-related deaths were obtained from Our World in Data, an official website for all COVID-19 (https://ourworldindata.org/coronavirus-source-data).

### 2.2. Methods

The zero-inflated Poisson (ZIP) and zero-inflated negative binomial (ZINB) autoregressive models proposed by Yang [14] and Yang et al. [15] were adapted to characterize the trend of COVID-19 deaths in Ghana, which is a discrete count time series with excess zeros.

Let {*Y*_*t*_}  denote the observed COVID-19 deaths, composed of discrete count data which is conditionally distributed as ZIP (*λ*_*t*_,  *ω*_*t*_), where *λ*_*t*_ is the intensity parameter of the baseline Poisson distribution and *ω*_*t*_ is the zero-inflation parameter. The zero-inflated Poisson autoregressive (ZIPA) has a probability distribution given by

(1) $$\begin{array}{c} P\left(Y_{t}|y_{t-1}=j\right)=\left\{\begin{array}{cc} \omega_{t}+\left(1-\omega_{t}\right)\text{exp}\left(-\lambda_{t}\right), & \text{if }j=0,\\ \left(1-\omega_{t}\right)\frac{\lambda_{t}^{j}\text{exp}\left(-\lambda_{t}\right)}{j!}, & \text{if }j=1,\;2,\;3,\;\ldots , \end{array}\right) \end{array}$$

where the intensity parameter *λ*_*t*_ and zero-inflation parameter *ω*_*t*_ are modelled as follows:

(2) $$\begin{array}{c} \log\;\lambda_{t}=X_{t-1}^{T}\beta , \end{array}$$

(3) $$\begin{array}{c} \log\left(\frac{\omega_{t}}{1-\omega_{t}}\right)=Z_{t-1}^{T}\gamma , \end{array}$$

where *β*=(*β*_1_,…,*β*_*p*_)^*T*^ and *γ*=(*γ*_1_,…,*γ*_*p*_)^*T*^ are the regression coefficients for the log-linear part (2) and logistic part (3), respectively. Vectors representing past explanatory variables which can incorporate functions of the lagged response series accounting for serial correlation are denoted by *X*_*t*−1_=(*x*_*t*−1,1_,…, *x*_*t*−1,*p*_)^*T*^ and *Z*_*t*−1_=(*z*_*t*−1,1_,…,*z*_*t*−1,*p*_)^*T*^. The conditional mean and variance of the ZIPA are

(4) $$\begin{array}{c} E\left(Y_{t}|y_{t-1}\right)=\lambda_{t}\left(1-\omega_{t}\right), \end{array}$$

(5) $$\begin{array}{c} \text{Var}\left(Y_{t}|y_{t-1}\right)=\left(1-\omega_{t}\right)\left(1+\lambda_{t}\omega_{t}\right)\lambda_{t}. \end{array}$$

Kedem and Fokianos [23] formulated the partial likelihood (PL) of the ZIPA as

(6) $$\begin{array}{c} \text{PL}=\prod_{t=1}^{n}f_{Y_{t}}\left(y_{t}|y_{t-1}\right). \end{array}$$

Substituting (1) into (6) yields

(7) $$\begin{array}{c} \text{PL}=\prod_{t=1}^{n}I\left(y_{t}=0\right)\left[\omega_{t}+\left(1-\omega_{t}\right)\text{exp}\left(-\lambda_{t}\right)\right]\times \prod_{t=1}^{n}I\left(y_{t}=1,\;2,\ldots \right)\left[\left(1-\omega_{t}\right)\frac{\lambda_{t}^{j}\text{exp}\left(-\lambda_{t}\right)}{j!}\right]. \end{array}$$

Also, when expressions for *λ*_*t*_ in (2) and *ω*_*t*_ in (3) are, respectively, put into the above expression, we have

(8) $$\begin{array}{c} \text{PL}=\prod_{t=1}^{n}I\left(y_{t}=0\right)\left[\frac{\exp\left(Z_{t-1}^{T}\gamma \right)}{1+\exp\left(Z_{t-1}^{T}\gamma \right)}+\frac{\exp\left[-\exp\left(X_{t-1}^{T}\beta \right)\right]}{1+\exp\left(Z_{t-1}^{T}\gamma \right)}\right]\times \;\prod_{t=1}^{n}I\left(y_{t}=1,2,\ldots \right)\left[\frac{1}{1+\exp\left(Z_{t-1}^{T}\gamma \right)}\left[\frac{{\left(\exp\left(X_{t-1}^{T}\beta \right)\right)}^{j}\exp\left[-\exp\left(X_{t-1}^{T}\beta \right)\right]}{j!}\right]\right]. \end{array}$$

Yang [14] and Yang et al. [15] developed the Expectation-Maximization (EM) algorithm in the partial likelihood framework to obtain parameter estimates and their standard errors.

Even though the ZIPA may correct for overdispersion [a phenomenon where Var(*Y*_*t*_*|y*_*t*−1_) > *E*(*Y*_*t*_*|y*_*t*−1_)] in discrete count time series data with excess zeros, we extended the ZIPA to zero-inflated negative binomial autoregressive (ZINBA) model which is well known for overdispersed data.

For the ZINBA, the probability distribution is given by

(9) $$\begin{array}{c} P\left(y_{t}|y_{t-1}=j\right)=\left\{\begin{array}{cc} \omega_{t}+\left(1-\omega_{t}\right){\left(\frac{k_{t}}{k_{t}+\lambda_{t}}\right)}^{k_{t}}, & \text{if }j=0,\\ \left(1-\omega_{t}\right)\frac{\Gamma \left(k_{t}+y_{t}\right)}{\Gamma \left(k_{t}\right)y_{t}!}{\left(\frac{k_{t}}{k_{t}+\lambda_{t}}\right)}^{k_{t}}{\left(\frac{\lambda_{t}}{k_{t}+\lambda_{t}}\right)}^{y_{t}}, & \text{if }j=1,2,3,\ldots , \end{array}\right) \end{array}$$

with *λ*_*t*_ and *ω*_*t*_ modelled as in (2) and (3) respectively. The dispersion parameter, *k*_*t*_, is modelled as

(10) $$\begin{array}{c} \log\;k_{t}=S_{t-1}^{T}\alpha , \end{array}$$

where *α*=(*α*_1_,…,*α*_*p*_)^*T*^ is the regression coefficients and *S*_*t*−1_=(*s*_*t*−1,1_,…, *s*_*t*−1,*p*_)^*T*^ is a vector of past explanatory variables.

The mean and variance expressions for the ZINBA are the same as that of the ZIPA stated in (4) and (5), respectively. As in the ZIPA, the PL of the ZINBA is the same as in (6).

Thus, substituting (7) into (6) gives the PL

(11) $$\begin{array}{c} PL=\overset{n}{\underset{t=1}{\prod }}I\left(y_{t}=0\right)\left[\omega_{t}+\left(1-\omega_{t}\right){\left(\frac{k_{t}}{k_{t}+\lambda_{t}}\right)}^{k_{t}}\right]\times \overset{n}{\underset{t=1}{\prod }}I\left(y_{t}=1,\;2,\ldots \right)\left[\left(1-\omega_{t}\right)\frac{\Gamma \left(k_{t}+y_{t}\right)}{\Gamma \left(k_{t}\right)y_{t}!}{\left(\frac{k_{t}}{k_{t}+\lambda_{t}}\right)}^{k_{t}}{\left(\frac{\lambda_{t}}{k_{t}+\lambda_{t}}\right)}^{y_{t}}\right]. \end{array}$$

When expressions for *k*_*t*_ in (10), *λ*_*t*_ in (2), and *ω*_*t*_ in (3), respectively, are substituted into the PL above, it yields

(12) $$\begin{array}{c} \text{PL}=\overset{n}{\underset{t=1}{\prod }}I\left(y_{t}=0\right)\left[\frac{\exp\left(Z_{t-1}^{T}\gamma \right)}{1+\exp\left(Z_{t-1}^{T}\gamma \right)}+\left(\frac{1}{1+\exp\left(Z_{t-1}^{T}\gamma \right)}\right){\left(\frac{\exp\left(S_{t-1}^{T}\alpha \right)}{\exp\left(S_{t-1}^{T}\alpha \right)+\exp\left(X_{t-1}^{T}\beta \right)}\right)}^{\exp\left(S_{t-1}^{T}\alpha \right)}\right]\times \overset{n}{\underset{t=1}{\prod }}I\left(y_{t}=1,\;2,\ldots \right)\left[\left(\frac{1}{1+\exp\left(Z_{t-1}^{T}\gamma \right)}\right)\frac{\Gamma \left(\exp\left(S_{t-1}^{T}\alpha \right)+y_{t}\right)}{\Gamma \left[\left(\exp\left(S_{t-1}^{T}\alpha \right)\right)\right]y_{t}!}{\left(\frac{\exp\left(S_{t-1}^{T}\alpha \right)}{\exp\left(S_{t-1}^{T}\alpha \right)+\exp\left(X_{t-1}^{T}\beta \right)}\right)}^{\exp\left(S_{t-1}^{T}\alpha \right)}{\left(\frac{\exp\left(X_{t-1}^{T}\beta \right)}{\exp\left(S_{t-1}^{T}\alpha \right)+\exp\left(X_{t-1}^{T}\beta \right)}\right)}^{y_{t}}\right]. \end{array}$$

Parameter estimates and their corresponding standard errors in the PL above can be obtained by employing the EM algorithm proposed by Yang [14] and Yang et al. [15].

The models were compared based on the Akaike Information Criterion (AIC; [23, 24]), Bayesian Information Criterion (BIC; [23, 25]), and Takeuchi Information Criterion (TIC; [26]). These metrics combine a measure of model fit, typically twice the negative log-partial likelihood, with a penalty for model complexity, expressed as a function of the number of parameters [12]. The AIC and BIC are computed by the expressions:

(13) $$\begin{array}{c} \text{AIC}=-2\;\log\text{PL}+2k,\\ \text{BIC}=-2\;\log\text{PL}+k\;\log\left(n\right), \end{array}$$

where *k* is the number of parameters in the model and *n* is the number of observations.

The TIC is calculated by the expression:

(14) $$\begin{array}{c} \text{TIC}=-2\;\log\text{PL}+2\text{tr}\left\{J_{n}I_{n}^{-1}\right\}, \end{array}$$

where *I*_*n*_^−1^ is the information matrix and *J*_*n*_ is given by

(15) $$\begin{array}{c} J_{n}=\sum_{t=1}^{n}\left\{\frac{\partial \;\log f_{Y_{t}}\left(y_{t}|y_{t-1}\right)}{\partial \theta }\right\}{\left\{\frac{\partial \;\log f_{Y_{t}}\left(y_{t}|y_{t-1}\right)}{\partial \theta }\right\}}^{T}, \end{array}$$

with *θ*=(*β*, *γ*)^*T*^.

Model fitting was done in *R* [27] using the “ZIM” and the countreg packages [14, 15, 28].

## 3. Results and Discussion

**Figure 2:**
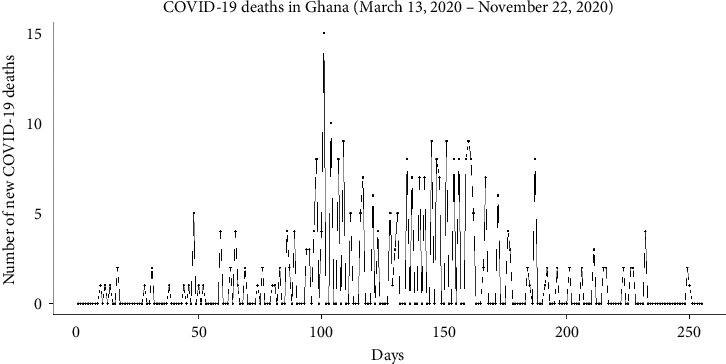
Trend of COVID-19 deaths in Ghana from 13^th^ March 2020 to 22^nd^ November 2020.

The trend of COVID-19 death in Ghana as illustrated in Figure 2 gives the impression of a general increase in the death toll from day zero (the day COVID-19 was first discovered in Ghana) to about day 160 after which there is an impression of a general decrease in the death toll to day 250 and beyond. The increase in the number of deaths was expected as the infection rate and active and severe cases continued to soar from day zero to day 150. The decrease in the number of active and severe cases amidst a rise in the infection rate could also be attributed to the decline in the number of deaths. However, a thorough investigation is needed to be carried out on the rise and fall of the deaths to ascertain what truly fuelled it.

**Figure 3:**
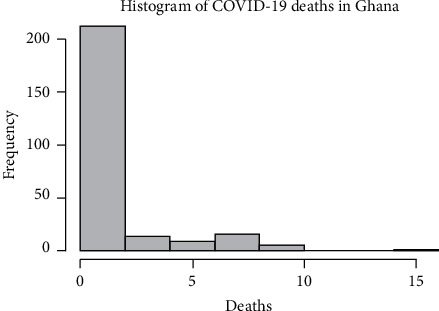
Histogram of COVID-19 deaths in Ghana from 13^th^ March 2020 to 22^nd^ November 2020.

As indicated by the histogram (Figure 3.), there is a higher proportion of zero counts (no deaths) per day making up 69.02% of the entire time series data. This is clearly an indication of zero inflation in the data. Even though infection rate continues to rise, the number of deaths being reported in most days ought to be looked into in order to confirm whether the majority of the COVID-19 patients in Ghana have developed resistance to the pandemic or they responded positive to care procedures meted to them at COVID-19 treatment centers.

In order to realize the most apt zero-inflated time series model to characterize the trend of our data, we fitted the ZIPA and ZINBA (Table 1). In the log-linear part of the models, the intercept of the ZIPA was significant at 0.05 significance level, but that of the ZINBA was not. However, the ZINBA has a smaller estimate (0.5821) compared to the estimate (1.1317) of the ZIPA. The count.lag and the trend were both not significant at 0.05. Fascinatingly, the count.lag of the ZIPA model had an increasing effect on the log of the expected number of COVID-19 deaths in Ghana, while it had a decreasing effect on that of the ZINBA model. The trend was also not significant in both the ZIPA model and the ZINBA model. Also, worth noting is the fact that even though the trend had an increasing effect on the log of expected number of COVID-19 deaths in both models, the effect is higher in the ZINBA than the ZIPA model.

On the logistic part of our models, the intercept of the ZIPA was significant with a higher estimate as compared to the nonsignificant intercept of the ZINBA with a smaller estimate. Just as in the log-linear part, the trend was not significant in the logistic part of the ZIPA and ZINBA models. In both models, the trend has an increasing effect on the log of the expected odds of the number of COVID-19 deaths in Ghana with the odds in the ZINBA model being higher than that of the ZIPA model.

The ZIPA model has higher AIC, BIC, and TIC values than the ZINBA model. For each of these criteria for assessing goodness of fit, there is a tart reduction from the value of the ZIPA to that of the ZINBA. This clearly shows that the ZINBA model may have corrected for more complexity in the data than the ZIPA model.

The test for overdispersion conducted (Table 2) had a score test of 8.3470 and a *P* value less than 0.0001. This means that the ZINBA model did well with respect to the overdispersion in our data.

Output from the dynamic zero-inflated Poisson autoregressive (DZIPA) and dynamic zero-inflated negative binomial autoregressive (DZINBA) models, with 200 replications, 100 iterations, and sample size of 200 in each model, are presented in Table 3. The zero-inflation parameter dwindled from the DZIPA (0.6148) to the DZINBA (0.6108). This could mean that the DZIPA may have detected more zeros in the data than the DZINBA. The standard deviation was higher in the DZIPA than in the DZINBA.

In the log-linear part of the models, the intercept is significant in the DZIPA model, but not significant in the DZINBA model. The trend was not significant in both the DZIPA model and the DZINBA model. Nevertheless, the trend had a decreasing effect on the log of the expected number of COVID-19 deaths in Ghana with the DZIPA model having a greater decreasing effect than the DZINBA model. We can deduce that the dynamic models generally forecast a decrease in the expected number of COVID-19 deaths in Ghana.

In respect of the autoregressive part, AR (1) was significant in the DZIPA model as well as the DZINBA model. Thus, we can deduce the DZIPA and the DZINBA models are both AR (1).

There was an unsubstantial increase in the AIC and BIC values from the DZIPA to the DZINBA. The TIC values, however, registered a quantum increase from the DZIPA model to the DZINBA model. Consequently, we can assert that the DZIPA has corrected for more complexity in the data than the DZINBA. With respect to the AIC, BIC, and TIC values, the DZIPA model outperformed the ZIPA model. Notwithstanding, the DZINBA outperformed the ZINBA only in terms of the AIC and BIC values while the opposite is true for the TIC value.

**Figure 4:**
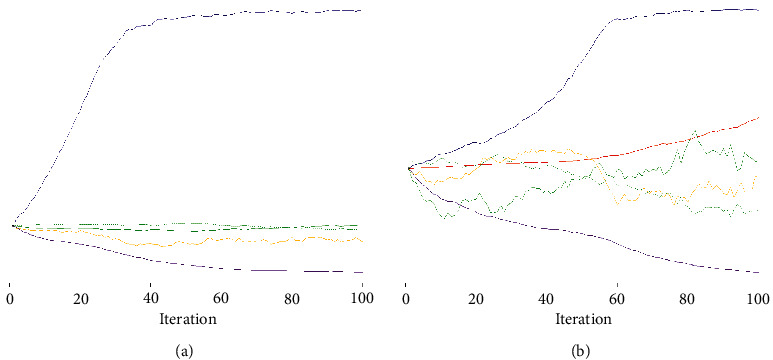
Trace plots from the DZIPA model (a) and DZINBA model (b) of COVID-19 deaths in Ghana.

Trace plots for the DZIPA and DZINBA models are presented in Figure 4. From the plots, we can see that the partial likelihood becomes progressively greater conspicuously preceding all others in time for several iterations and then maintains stability as the estimated parameters become very close to the maximum likelihood estimator [14, 15].

**Figure 5:**
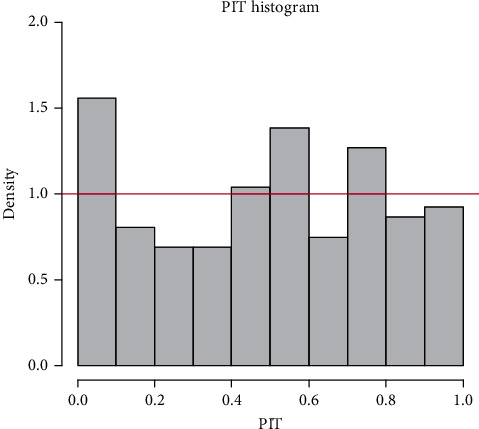
Probability integral transform (PIT) histogram for the ZINBA model.

Figure 5 points to a probability integral transform (PIT) histogram [29], which appears to approach uniformity. The horizontal line depicts the count that each of the bins would have if the histogram was perfectly uniform. Hence, the probabilistic calibration of the fitted ZINBA model is sufficient.

**Figure 6:**
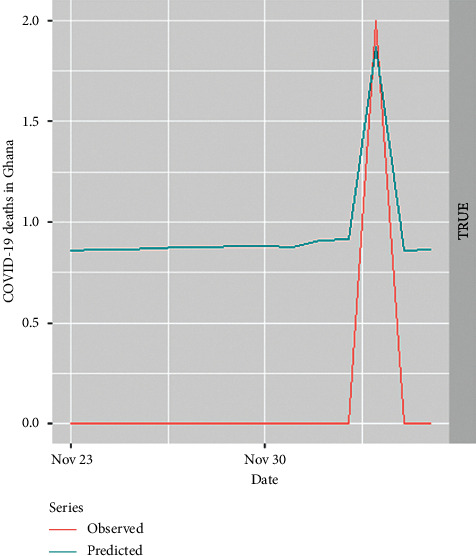
Out-of-sample model validation.

Time series of the observed daily new deaths of COVID-19 from 23^rd^ November 2020 to 6^th^ December 2020 and predicted daily new deaths based on the fitted ZINBA model are shown in Figure 6. It is observed that the overall trend of the two curves is similar, and the values themselves are very close in some cases. This is an indication of a good predictive model.

## 4. Conclusion

We observed that Ghana's COVID-19 daily death count, from the very first day the pandemic was discovered in the country, is inflated with zeros (no deaths). This excessive number of zeros lead to overdispersion.

The trend of COVID-19 deaths per day in Ghana is characterized by a general increase from the onset of the pandemic in the country to about day 160 after which there is a general decrease onwards. The continuous decrease in the death toll amidst rise in daily infections and continuous disregard of safety protocols recently ought to be investigated.

We fitted a zero-inflated Poisson autoregressive model and zero-inflated negative binomial autoregressive model to the data in the partial-likelihood framework. The zero-inflated negative binomial autoregressive model outperformed the zero-inflated Poisson autoregressive model. We further obtained dynamic versions of the zero-inflated models. The dynamic zero-inflated Poisson autoregressive model, however, performed better than the dynamic negative binomial autoregressive model. Both dynamic models predicted an AR (1).

The predicted new deaths based on the ZINBA model showed that Ghana's COVID-19 deaths per day will rise sharply few days after 30^th^ November 2020 and drastically fall just like that of the observed data.

## Figures and Tables

**Table 1:** ZIPA and ZINBA models estimate with corresponding standard errors (s.e.) for COVID-19 deaths in Ghana.

	ZIPA		ZINBA	
	Log-linear		Log-linear	
Coefficients	Estimate (s.e.)	Pr (>│*z*│)	Estimate (s.e.)	Pr (>│*z*│)
Intercept	1.1317 (0.1425)	<0.0001	0.5821 (0.4654)	0.2210
Count.lag1	0.0580 (0.1309)	0.6578	−0.0391 (0.2581)	0.8797
Trend	1.8618 (0.9858)	0.0590	4.9987 (3.2218)	0.1208
	Logistic		Logistic	
Intercept	0.6342 (0.2774)	0.0223	0.0896 (0.5284)	0.8654
Trend	1.0210 (1.8822)	0.5875	3.0297 (2.8942)	0.2952
Dispersion, *k*_*t*_	—	—	1.5353	—
Goodness of fit				
AIC	720.7728	—	681.6503	—
BIC	738.4595	—	702.8743	—
TIC	729.1185	—	683.6137	—

**Table 2:** Test for overdispersion.

*H*_0_: ZIPA	
*H*_1_: ZINBA	
Score.test	8.3470
*P* value	<0.0001

**Table 3:** Dynamic zero-inflated Poisson autoregressive (DZIPA) model and dynamic negative binomial autoregressive (DZINBA) model estimate with corresponding standard errors (s.e.) for COVID-19 deaths in Ghana.

	DZIPA		DZINBA	
	Log-linear		Log-linear	
Coefficients	Estimate (s.e.)	Pr (>│*z*│)	Estimate	Pr (>│*z*│)
Intercept	1.1012 (0.5469)	0.0441	0.8066 (1.2224)	0.5093
Trend	−1.3129 (3.9978)	0.7426	−0.3020 (7.4510)	0.9677

Autoregressive				
AR1	0.9735 (0.0172)	<0.0001	0.9618 (0.0291)	<0.0001
Zero-inflation	0.6148	—	0.6108	—
Standard deviation	0.1908	—	0.2285	—
*N*	200	—	200	—
*R*	200	—	200	—
Number of iterations	100	—	100	—
Dispersion, *k*_*t*_	—	—	11.3647	—

Goodness of fit				
AIC	629.1376	—	636.9148	—
BIC	646.8336	—	658.1624	—
TIC	722.0860	—	1023.5070	—

## Data Availability

The data used are made up of daily COVID-19 death count in Ghana from 13^th^ March 2020 to 22^nd^ November 2020 from Our World in Data, an official website for all COVID-19–related deaths (https://ourworldindata.org/coronavirus-source-data).
